# First report of mitogenome of *Subclytia rotundiventris* (Diptera, Tachinidae) yielded by next-generation sequencing

**DOI:** 10.1080/23802359.2019.1661297

**Published:** 2019-09-06

**Authors:** Wenya Pei, Liping Yan, Nan Yang, Chuntian Zhang, Changyan Zheng, Jun Yang, Dong Zhang

**Affiliations:** a School of Nature Conservation, Beijing Forestry University, Beijing, China;; b Administration Department of Beijing, Baihuashan National Nature Reserve, Beijing, China;; c College of Life Science, Shenyang Normal University, Shenyang, China

**Keywords:** Phasiinae, *Subclytia rotundiventris*, mitochondrial genome, phylogeny

## Abstract

The mitochondrial genome of *Subclytia rotundiventris* (Fallén, 1820) belongs to the subfamily Phasiinae of Tachinidae, was obtained using a next-generation sequencing approach. This 15,574 bp mitogenome consists of 22 transfer RNA genes, 13 protein-coding genes, 2 ribosomal RNA genes, and 1 noncoding control region. Our results strongly supported the monophyly of Tachinidae. It also indicated that the monophyly of the Dexiinae, Tachininae, Phasiinae, and Exoristinae is consistently fully supported and clustered as (Dexiinae (Tachininae (Phasiinae + Exoristinae))).

Tachinid flies are the most diverse group of parasitoid Diptera with over 8500 described species and many species act as important natural enemies of herbivorous insects in both natural and managed terrestrial ecosystems, especially forests (Stireman and Singer 2003; Stireman et al. 2006; O’Hara et al. 2009; O’Hara and Cerretti 2016). Due to its various morphological features, entomologists face great challenges to identify tachinid flies and the phylogenetic relationships within the Tachinidae are unclear at the higher levels (Meier et al. 2006; O'Hara 2013). Molecular systematics is beginning to show promise in the elucidation of tachinid relationships, but there have been few studies to date. Here, we sequenced mitochondrial genome of *Subclytia rotundiventris* (Fallén, 1820), the representative of subfamily Phasiinae for further research.

The adult specimens of *Subclytia rotundiventris* were collected on 16 June 2017 from Baihuashan National Nature Reserve of Beijing, China (39. 836403 N, 115. 578186 E). The specimens were deposited at the Museum of Beijing Forestry University, Beijing, China (Accession number: BFU14C6-5). The genomic DNA was extracted from the adult’s muscle tissues of the thorax using the DNeasy Blood and Tissue kit (QIAGEN Sciences, Valencia, CA, USA). The genomic DNA was pooled with other insect species and sequenced using the Illumina Nova6000 (PE150, Illumina, San Diego, CA, USA) platform. DNA reads were assembled employing idba_ud implemented with IDBA-1.1.1 (Peng et al. 2012). Mitogenome was pulled out with COI as bait sequence (Ding et al. 2019) and annotated referring Zhang et al. (2016).

The mitochondrial genome of *Subclytia rotundiventris* (GenBank accession No. MN199029) is 15,574 bp in length and contains 22 transfer RNA genes, 13 protein-coding genes (PCGs), 2 ribosomal RNA genes, and 1 noncoding control region. The nucleotide composition is biased towards A and T (80.06%) than other calyptratae (Zhang et al. 2016; Li et al. 2017; Hou et al. 2019), with 41.01% of A, 39.05% of T, 11.52% of C, and 8.42% of G. Most of the 13 PCGs used ATN as the start codon (ATG for COII, ATP6, COIII, ND4, ND4L and CYTB; ATT for ND2, ND5, and ND6; ATA for ND3 and ND1; ATT for ATP8), except that COI begins with codon TCG. The stop codon TAA is assigned to most of the PCGs (ND2, COI, ATP8, ATP6, COIII, ND4L, ND6, and CYTB), whereas incomplete stop codon T is used by three PCGs (COII, ND5, and ND4), ND3 and ND1 stop with the codon TAG.

Complete mitochondrial genome sequences of the other genera of Tachinidae available from GenBank were mined for phylogenomic analysis. Bayesian phylogenetic analysis was performed on the basis of the dataset containing sequences of 13 PCGs and 2 rRNAs (Figure 1). According to the phylogenetic outcome, the outgroups *Lucilia sericata* (Calliphoridae) and *Sarcophaga crassipalpis* (Sarcophagidae) form a clade diverged from Tachinidae clades. Our results strongly supported the monophyly of Tachinidae. It also indicated that the monophyly of the Dexiinae, Tachininae, Phasiinae, and Exoristinae is consistently fully supported and clustered as (Dexiinae (Tachininae (Phasiinae + Exoristinae))).

**Figure 1.**
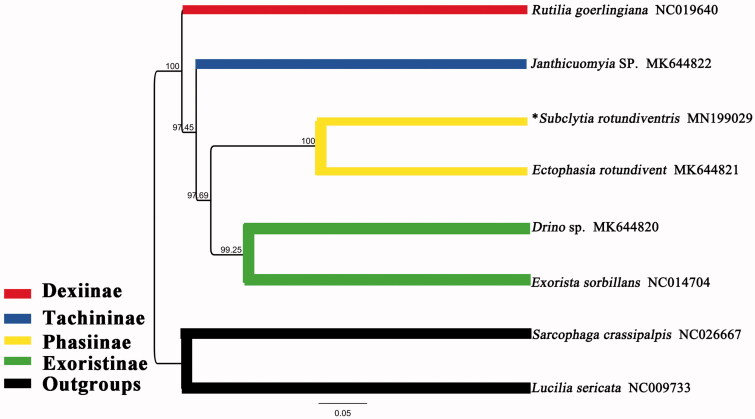
Bayesian phylogenetic tree of eight species which consists of six Tachinidae species and two outgroups. *indicates this study.
